# mRNA technology as one of the promising platforms for the SARS-CoV-2 vaccine development

**DOI:** 10.18699/VJ20.676

**Published:** 2020-11

**Authors:** A.A. Ilyichev, L.A. Orlova, S.V. Sharabrin, L.I. Karpenko

**Affiliations:** State Research Center of Virology and Biotechnology “Vector”, Koltsovo, Novosibirsk region, Russia; State Research Center of Virology and Biotechnology “Vector”, Koltsovo, Novosibirsk region, Russia; State Research Center of Virology and Biotechnology “Vector”, Koltsovo, Novosibirsk region, Russia; State Research Center of Virology and Biotechnology “Vector”, Koltsovo, Novosibirsk region, Russia

**Keywords:** coronavirus, SARS-CoV-2, COVID-19, mRNA-vaccines, коронавирус, SARS-CoV-2, COVID-19, мРНК-вакцины

## Abstract

After the genome sequence of SARS-CoV-2 (Severe acute respiratory syndrome-related coronavirus 2) was published and the number of infected people began to increase rapidly, many global companies began to develop a vaccine. Almost all known approaches to vaccine design were applied for this purpose, including inactivated viruses, mRNA and DNA-vaccines, vaccines based on various viral vectors, synthetically generated peptides and recombinant proteins produced in cells of insects and mammals. This review considers one of the promising vaccine platforms based on messenger RNA. Until recent years, mRNA-vaccination was out of practical implementation due to high sensitivity to nuclease degradation and consequent instability of drugs based on mRNA. Latest technological advances significantly mitigated the problems of low immunogenicity, instability, and difficulties in RNA-vaccine delivery. It is worth noting that mRNA-vaccines can efficiently activate both components of the immune system, i. e. T-cell and humoral responses. The essential advantage of mRNAvaccines includes fast, inexpensive, scalable and uniform production providing a large output of desirable products in vitro. Synthesis and purification processes significantly simplify the process technology of mRNA drugs with injectable purity. Thus, mRNA production via in vitro transcription is more advantageous as compared with DNA-vaccines since it is a chemical process without the use of cells. mRNA techniques make it possible to pass all the phases of vaccine development much faster in comparison with the production of vaccines based on inactivated viruses or recombinant proteins. This property is critically important when designing vaccines against viral pathogens as the main problem of disease control includes a time gap between an epidemic and vaccine development. This paper discusses studies on the development of vaccines against coronaviruses including SARS-CoV-2 with special attention to the mRNA technique.

## Abstract (ru)

После того как была опубликована последовательность генома SARS-CoV-2 (Severe acute respiratory syndrome-related coronavirus 2), а количество заболевших стало стремительно возрастать, многие глобальные компании начали разработку вакцины от данного вируса. Для создания вакцины задейство- ваны практически все известные на данный момент способы – это вакцины на основе инактивированного вируса, мРНК и ДНК, вирусных векторов, синтетических пептидов и рекомбинантных белков, произведен- ных в клетках насекомых и млекопитающих. В обзоре рассматривается одна из перспективных вакцинных платформ, созданная на основе матричной РНК (мРНК). До недавнего времени мРНК-вакцинация не рас- сматривалась с практической точки зрения в силу высокой чувствительности к нуклеазной деградации и, как следствие, нестабильности препаратов на основе мРНК. Последние технологические достижения в значительной степени преодолели проблемы низкой иммуногенности, нестабильности и трудности до- ставки РНК-вакцин. Важно отметить, что мРНК-вакцины способны эффективно активизировать оба звена иммунитета – как Т-клеточный, так и гуморальный ответы. Существенным преимуществом мРНК-вакцин является быстрое недорогое масштабируемое и однотипное производство, обеспечивающее высокие выходы желаемого продукта в условиях in vitro. После синтеза и процедуры очистки технологически зна- чительно проще добиться получения препарата мРНК инъекционной чистоты. Таким образом, производ- ство мРНК путем транскрипции in vitro предпочтительнее в сравнении с производством ДНК-вакцин, так как в действительности является химическим процессом без использования клеток. По сравнению с про- изводством вакцин на основе инактивированного вируса или рекомбинантного белка мРНК-технологии позволяют гораздо быстрее пройти все этапы разработки. Этот параметр имеет первостепенное значе- ние для создания препаратов против вирусных патогенов, основной проблемой борьбы с которыми яв- ляется временной разрыв между эпидемией и разработкой вакцины. В данном обзоре мы обсуждаем работы, связанные с разработкой вакцины против коронавирусов, включая SARS-CoV-2, с акцентом на технологии мРНК.

## Article

After the first sequence of the severe acute respiratory syndrome- related coronavirus 2 (SARS-CoV-2) genome was published by Chinese researchers in January 2020, many scientific organisations and pharmaceutical companies began developing a vaccine against SARS-CoV-2 (Zhou P. et al., 2020). For this purpose, almost all known approaches for vaccine design were applied, including inactivated viruses, mRNA and DNA vaccines, vaccines based on various viral vectors, recombinant proteins produced in cells of insects and mammals, and synthetic peptide-based vaccines.

In this article we will consider the advantages of mRNA vaccines.

SARS-CoV-2 belongs to the family of Coronaviridae, which also includes dangerous viruses such as severe acute respiratory syndrome-related coronavirus (SARS-CoV) and Middle East respiratory syndrome-related coronavirus (MERS-CoV)^1^. These viruses have a single-stranded RNA genome about 30 kb in size, which is the largest known RNA virus genome. The complete genomes of SARS-CoV-2 and SARS-CoV have very high homology, suggesting that the mechanisms of entry of these viruses into human cells are similar (Zhou P. et al., 2020). The viral envelope consists of a lipid bilayer in which the structural proteins of the membrane (M), envelope (E), and spike (S) are fixed. The nucleocapsid (N) protein, together with the viral RNA genome, form a helical core located within the viral envelope. The ratio of S : E : M : N proteins corresponds to 20 : 1 : 300 : 100. The globular part of the S protein contains many dominant antigenic epitopes involved in the mechanisms of the humoral and cellular immune response (Zhou Y. et al., 2018). The S protein plays a crucial role in the attachment of coronaviruses to a cell surface receptor and, consequently, entry of the virus into the cell. For SARS-CoV-2 and SARS-CoV, the S protein receptor is angiotensin converting enzyme 2 (ACE2). Therefore, the S protein is considered the most suitable target for vaccine development (He et al., 2006).

WHO, https://www.who.int



Research on the development of vaccines against the SARSCoV (2002) and MERS-CoV (2012) viruses were carried out but were never completed. This is partly due to the fact that the SARS-CoV epidemic lasted for a relatively short time, about 15 months, and the last case was recorded in June 2003. In total, more than 8,000 people were infected (Kim et al., 2019). Since 2004, no cases of SARS-CoV infection have been reported. The MERS-CoV virus has caused sporadic outbreaks in various countries, with the most recent case detected in February 2020 in Qatar (de Wit et al., 2020). However, this existing research on the development of a vaccine against these viruses has provided an important understanding of the mechanisms that mediate the induction of a protective immune responses against SARS-CoV and MERS-CoV, and these findings are being taken into account by the designers of SARS-CoV-2 vaccines.

A number of studies have demonstrated that antibodies generated against the SARS-CoV S protein can protect laboratory animals from virus infection (Yang et al., 2004). However, the humoral response was short-lived in people who had SARS-CoV infection (Tang et al., 2011). At the same time, a virus-specific T cell response was recorded up to 11 years after infection (Ng et al., 2016). These data highlight the importance of the T cell response, which should be taken into account when developing effective immunogens that can stimulate cytotoxic and helper responses against SARS-CoV-2.

Different approaches were used to develop vaccines against SARS-CoV and MERS-CoV, including vaccines based on inactivated virus, viral vectors, recombinant proteins, peptides, and DNA and RNA vaccines. The same approaches are being used to create a vaccine against SARS-CoV-2 now. According to the website of the World Health Organization (WHO), on August 13, 2020, more than 100 SARS-CoV-2 vaccine prototypes were being developed (Draft landscape of COVID-19 candidate vaccines, 2020). Such a variety of prototypes in the first stages is understandable as there is no universal solution to the problem at the moment.

It should be noted that more than 10 vaccines from this list have been developed on the basis of mRNA, a rapidly developing technology in recent years (Table).

**Table 1.**
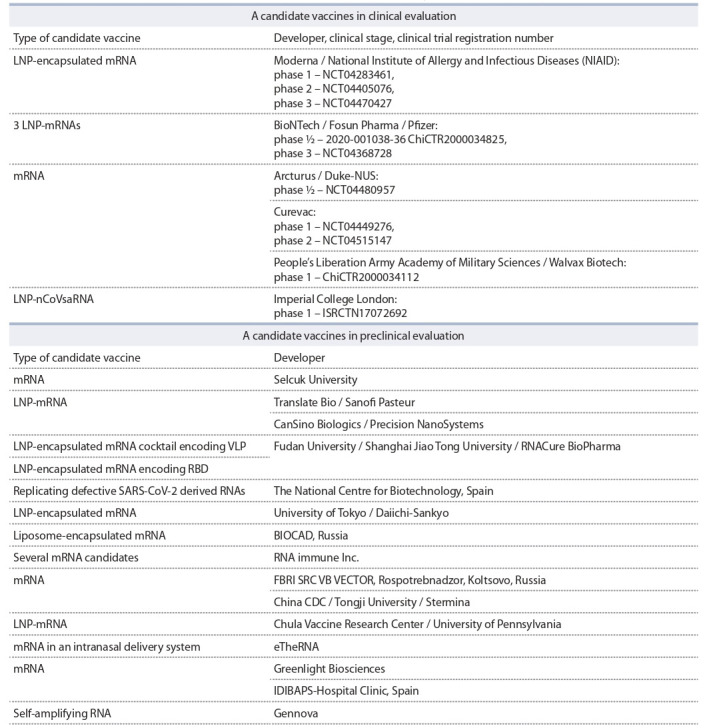
List of mRNA-based vaccines under development against COVID-19 registered by WHO as of October 2, 2020

Among the developers of mRNA vaccines are such research centres and companies as Moderna Inc. (USA), CureVac and BioNTech (Germany), Oxford University (UK), CanSino Biologics Inc. (China), VIDO-InterVac (Canada), and BIOCAD (Russia).

Until recently, the development of preventive and therapeutic RNA-based vaccines has been fraught with problems due to mRNA instability and inefficient delivery. Progress in this area can be attributed to advances in mRNA synthesis technology, optimisation of the secondary structure of mRNA and the cap structure, increasing resistance to RNA degradation by nucleases by the inclusion of modified nucleosides such as pseudouridine and 5-methylcytidine, and improvements in methods for RNA purification and delivery (Pardi et al., 2018). The necessary enzymes and ingredients are currently commercially available, which allows the production of mRNA in the necessary quantities for mass vaccination of the population. In recent years, a number of mRNA vaccines have been developed and tested against a variety of infectious diseases (influenza, rabies virus, Zika virus, HCV, HMV, etc.) and several types of cancer. These vaccines have shown promising results in both animal and human models (Pardi et al., 2018). It is important to note that mRNA vaccines can effectively activate both parts of the immune response – both T cells and humoral responses (Zhang et al., 2019).

RNA-based vaccines can be divided into two types: nonreplicating mRNAs and self-amplifying RNAs (Iavarone et al., 2017). Non-replicating RNA vaccines are composed of the mRNA that encodes the amino acid sequence of the target protein (the immunogen) together with all necessary elements for the translation process. Self-amplifying RNA vaccines are replicons constructed from positive single-stranded RNA viruses, such as alpha viruses and flaviviruses. Such replicons usually consist of two parts: one of them encodes nonstructural proteins that carry out viral RNA replication, while the other encodes the target protein (immunogen) (Iavarone et al., 2017). Self-amplifying RNA vaccines are characterised by higher and longer expression of the target gene compared to non-replicating analogues. However, these RNA replicons are very sensitive to the size of the embedded target. In addition, a large vector size (about 10 kb) may limit the efficiency of cell internalisation (Schwendener, 2014).

A schematic diagram of the mRNA-based vaccine and the mechanism of antigen presentation are presented in Figure. After the mRNA enters the cell, it is translated through the cellular mechanism of protein synthesis. Translation can occur both on ribosomes located in the cytoplasm in free form and on ribosomes associated with the membranes of the endoplasmic reticulum. There are some variants of antigen presentation pathway (see Figure).

**Fig. 1.**
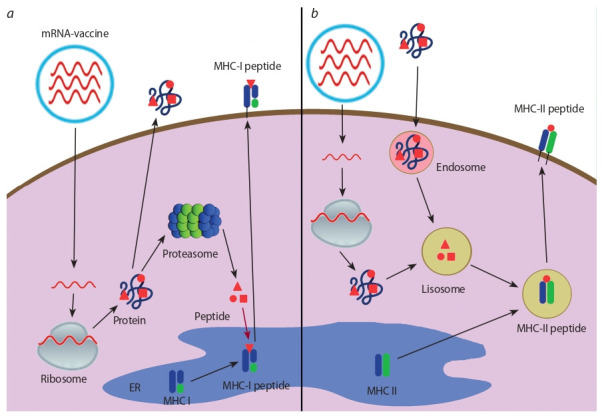
Messenger RNA vaccine and antigen presentation. a – muscle cells; b – specialised antigen-presenting cells.

- The protein enters the proteasome where it undergoes processing and is cleaved into small peptides (epitopes). These peptides are then transferred to the lumen of the endoplasmic reticulum by transporter associated with antigen processing proteins, where they bind to MHC class I molecules. The resulting complex in the vesicle is directed to the plasma membrane of the cell and exposed on the cell surface, where it is recognised by the CD8+ receptors of cytotoxic lymphocytes, stimulating a specific cytotoxic cellular response.
- The protein is enclosed in vesicles called lysosomes, where the antigen is cleaved into peptide fragments by lysosomeassociated enzymes (acid proteases). The lysosome merges with the vesicle that carries the MHC class II molecule. Within this structure, an epitope complex with MHC II is formed. The complex is transported to the cell membrane and brought to the surface, where it is recognised by CD4^+^ lymphocyte receptors. As a result, both the T-helper response and humoral immunity (activation of B lymphocytes) are activated.
- The protein can be secreted by the cell, activate B lymphocytes, and induce the humoral immune response.

mRNA-based vaccines have a range of useful features in comparison to other types of vaccines, such as classic vaccines (based on a live attenuated or inactivated virus), and protein and DNA vaccines. Firstly, mRNA vaccines are known for their safety. mRNAs are non-infectious, unlike classical viral vaccines, and they have low reactogenicity. An important point of difference from classical vaccines is the lack of strict temperature control required for storage of drugs based on mRNA. Currently, most vaccines need to be transported and stored under cold chain conditions, which causes serious challenges for their delivery to remote regions. The lyophilised mRNA vaccine can be stable at 5–25 °C for 36 months, which makes it possible to eliminate this disadvantage. Unlike a DNA vaccine, mRNA cannot integrate into the cell genome and cause mutations. Thus, there is no risk of insertion of foreign genetic information into the patient’s genome. mRNA, being a minimal genetic vector, does not lead to the antivector immune response observed when using viral carriers. Thus, it can be used for immunisation multiple times.

Messenger RNA is subject to physiological destruction as a result of processes occurring in the cell. Its half-life can be regulated by modifications of the RNA sequence and its delivery method (Kauffman et al., 2016; Guan, Rosenecker, 2017).

A significant advantage of mRNA vaccines is its fast, inexpensive, scalable, and uniform production, which provides high yields of the desired product in vitro. It does not require the cultivation of bacteria or the use of cell cultures or chicken embryos, which are necessary for most types of antiviral vaccines. All that is required for the production of an mRNA vaccine is a DNA matrix that carries the target gene under the control of the T7 phage promoter, in addition to a set of enzymes for matrix synthesis. After the synthesis and purification procedure, it is technologically much easier to obtain an injectable mRNA preparation than a DNA vaccine. Thus, the production of mRNA by in vitro transcription is more attractive than the production of DNA vaccines because it is essentially a chemical process that does not require the use of cells (Liu et al., 2019).

An important but non-optimised part of mRNA vaccine technology is its delivery. To perform its task, the mRNA must enter the cell’s cytoplasm where the protein encoded by it can be translated. A range of mRNA delivery methods have been described, including administration of the vaccine by electroporation, injection into muscles, lymph nodes, or directly into organs, or administration intranasally, rectally, or orally (Gómez-Aguado et al., 2020; Wadhwa et al., 2020). Messenger RNA vaccination is also hindered by its degradation by various extracellular ribonucleases, which are abundant in tissues and in the intercellular space (Houseley, Tollervey, 2009).

A variety of approaches have been used to deliver and protect mRNA from degradation by nucleases. Lipid nanoparticles are currently one of the most commonly used means of delivering mRNA. Standard lipid nanoparticles consist of four components: cationic lipid, cholesterol, auxiliary phospholipids, and polyethylene glycol. Cationic polymer materials such as dendrimers and polyethylenimine, among others, are promising materials for facilitating the delivery of nucleic acids. Gene gun and electroporation techniques can also be used (Capasso et al., 2018; Kowalski et al., 2019). It is possible to increase the stability of the mRNA molecule by including nucleotide analogues such as pseudouridine, methylpseudouridine, and methylcytodine. However, sometimes the use of such modifications leads to a decrease in the efficiency of translation.

An important advantage of mRNA vaccine technology compared to the production of vaccines based on inactivated virus or recombinant protein is the ability to quickly pass all stages of its development. This quality is very important for the development of vaccines against viral pathogens, the main problem of which is the time gap between the start of the epidemic and the development of the vaccine. To prevent outbreaks of newly emerging and rapidly evolving pathogens, the speed of response to the pandemic with the creation of a preventive vaccine is of paramount importance. It has recently been shown that by using a synthetic biology approach including bioinformatics, a prototype vaccine against a target viral pathogen in mRNA format can be developed in a week (Rauch et al., 2018).

The developers of the mRNA vaccine against SARS-CoV-2 in the United States (Moderna Inc. together with the National Institute of Allergy and Infectious Diseases, NIAID) created a prototype of the mRNA-1273 vaccine in an unprecedentedly short time^2^. It took just 63 days from the selection of the viral sequence to the development of the vaccine for the first phase of clinical testing, in which 45 volunteers were given three different doses over 6 weeks to obtain initial safety data 

Moderna press-release: https://investors.modernatx.com/news-releases/news-release-details/moderna-ships-mrna-vaccine-against-novel-coronavirusmrna-1273



The most interesting thing is that the mRNA-1273 vaccine did not pass all of the preclinical tests before it was used in the first phase of clinical trials after proving its specific activity.

What prompted the researchers from the United States to choose this approach? Firstly, the development of this vaccine was based on previous projects by the developers to create vaccines against other types of coronavirus, such as SARS and MERS, which were unfortunately never completed. There are also dozens of studies on the use of mRNAs as therapeutic vaccines for the treatment of cancer, with no significant adverse reactions to the vaccine observed (Sebastian et al., 2014; Pardi et al., 2020).

In conclusion, we can say with some confidence that RNAbased vaccines can be effective against pandemics caused by viruses, including SARS-CoV-2, as this approach offers a relatively simple and fast solution for newly emerging and returning viral pathogens.

## Conflict of interest

The authors declare no conflict of interest.

## References

Capasso P.U., Kaczmarek J.C., Fenton O.S., Anderson D.G. Poly(betaamino
ester)-co-poly(caprolactone) Terpolymers as Nonviral Vectors
for mRNA Delivery In Vitro and In Vivo. Adv. Healthc. Mater.
2018;7(14):e1800249. DOI 10.1002/adhm.201800249.

de Wit E., Feldmann F., Cronin J., Jordan R., Okumura A., Thomas T.,
Scott D., Cihlar T., Feldmann H. Prophylactic and therapeutic remdesivir
(GS-5734) treatment in the rhesus macaque model of MERSCoV
infection. Proc. Natl. Acad. Sci. USA. 2020;117(12):6771-
6776. DOI 10.1073/pnas.1922083117.

Draft landscape of COVID-19 candidate vaccines. World Health Organization.
2020. Available at: https://www.who.int/who-documentsdetail/
draft-landscape-of-covid-19-candidate-vaccines.

Gómez-Aguado I., Rodríguez-Castejón J., Vicente-Pascual M., Rodríguez-
Gascón A., Solinís M.A., Del Pozo-Rodríguez A. Nanomedicines
to Deliver mRNA: State of the Art and Future Perspectives.
Nanomaterials (Basel ). 2020;10(2):364. DOI 10.3390/nano
10020364.

Guan S., Rosenecker J. Nanotechnologies in delivery of mRNA therapeutics
using nonviral vector-based delivery systems. Gene Therapy.
2017;24(3):133-143. DOI 10.1038/gt.2017.5.

He Y., Li J., Heck S. Lustigman S., Jiang S. Antigenic and immunogenic
characterization of recombinant baculovirus-expressed severe
acute respiratory syndrome coronavirus spike protein: Implication
for vaccine design. J. Virol. 2006;80:5757-5767. DOI 10.1128/
JVI.00083-06.

Houseley J., Tollervey D. The Many Pathways of RNA Degradation.
Cell. 2009;136(4):763-776. DOI 10.1016/j.cell.2009.01.019.

Iavarone C., O’hagan D.T., Yu D., Delahaye N.F., Ulmer J.B. Mechanism
of action of mRNA-based vaccines. Expert Rev. Vaccines.
2017;16(9):871-881. DOI 10.1080/14760584.2017.1355245.

Kauffman K.J., Webber M.J., Anderson D.G. Materials for non-viral
intracellular delivery of messenger RNA therapeutics. J. Control.
Release. 2016;240:227-234. DOI 10.1016/j.jconrel.2015.12.032.

Kim J.H., Kang M., Park E, Chung D.R., Kim J., Hwang E.S. A Simple
and Multiplex Loop-Mediated Isothermal Amplification (LAMP)
Assay for Rapid Detection of SARS-CoV. Biochip J. 2019;13(4):
341-351. DOI 10.1007/s13206-019-3404-3.

Kowalski P.S., Rudra A., Miao L., Anderson D.G. Delivering the
Messenger: Advances in Technologies for Therapeutic mRNA Delivery.
Mol. Ther. 2019;27(4):710-728. DOI 10.1016/j.ymthe.2019.
02.012.

Liu M.A. A comparison of plasmid DNA and mRNA as vaccine
technologies. Vaccines. 2019;7(2):37. DOI 10.3390/vaccines702
0037.

Ng O.-W., Chia A., Tan A.T., Jadi R.S., Leong H.N., Bertoletti A.,
Tan Y.-J. Memory T cell responses targeting the SARS coronavirus
persist up to 11 years post-infection. Vaccine. 2016;34(17):2008-
2014. DOI 10.1016/j.vaccine.2016.02.063.

Pardi N., Hogan M.J., Porter F.W., Weissman D. mRNA vaccines – a
new era in vaccinology. Nat. Rev. Drug Discov. 2018;17(4):261-279.
DOI 10.1038/nrd.2017.243.

Pardi N., Hogan M.J., Weissman D. Recent advances in mRNA vaccine
technology. Curr. Opin. Immunol. 2020;65:14-20. DOI 10.1016/
j.coi.2020.01.008.

Rauch S., Jasny E., Schmidt K.E., Petsch B. New vaccine technologies
to combat outbreak situations. Front. Immunol. 2018;9:1963. DOI
10.3389/fimmu.2018.01963.

Schwendener R.A. Liposomes as vaccine delivery systems: A review
of the recent advances. Ther. Adv. Vaccines. 2014;2(6):159-182. DOI
10.1177/2051013614541440.

Sebastian M., Papachristofilou A., Weiss C., Früh M., Cathomas R.,
Hilbe W., Wehler T., Rippin G., Koch S.D., Scheel B., Fotin-Mleczek
M., Heidenreich R., Kallen K.-J., Gnad-Vogt U., Zippelius A.
Phase Ib study evaluating a self-adjuvanted mRNA cancer vaccine
(RNActive®) combined with local radiation as consolidation and
maintenance treatment for patients with stage IV non-small cell
lung cancer. BMC Cancer. 2014;14:748. DOI 10.1186/1471-2407-
14-748.

Tang F., Quan Y., Xin Z.T., Wrammert J., Ma M.-J., Lv H., Wang T.-B.,
Yang H., Richardus H.J., Liu W., Cao W.-Ch. Lack of peripheral
memory B cell responses in recovered patients with severe acute
respiratory syndrome: A six-year follow-up study. J. Immunol. 2011;
86(12):7264-7268. DOI 10.4049/jimmunol.0903490.

Wadhwa A., Aljabbari A., Lokras A., Foged C., Thakur A. Opportunities
and challenges in the delivery of mRNA-based vaccines.
Pharmaceutics. 2020;12(2):102. DOI 10.3390/pharmaceutics1202
0102.

Yang Z.Y., Kong W.P., Huang Y., Roberts A., Murphy B.R, Subbarao K.,
Nabel G.J. A DNA vaccine induces SARS coronavirus neutralization
and protective immunity in mice. Nature. 2004;428(6982):561-564.
DOI 10.1038/nature02463.

Zhang C., Maruggi G., Shan H., Li J. Advances in mRNA vaccines
for infectious diseases. Front. Immunol. 2019;10:594. DOI 10.3389/
fimmu.2019.00594.

Zhou P., Yang X.-L., Wang X.-G., Hu B., Zhang L., Zhang W.,
Si H.- R, Zhu Y., Li B., Huang C.-L., Chen H.-D., Chen J., Luo Y.,
Guo H., Jiang R.-D., Liu M.-Q., Chen Y., Shen X.-R., Wang X.,
Zheng X.- S., Zhao K., Chen Q.-J., Deng F., Liu L.-Li., Yan B.,
Zhan F.-X., Wang Y.-Y., Xiao G.-F., Shi Z.-L. A pneumonia outbreak
associated with a new coronavirus of probable bat origin. Nature.
2020;579(7798):270-273. DOI 10.1038/s41586-020-2012-7.

Zhou Y., Jiang S., Du L. Prospects for a MERS-CoV spike vaccine.
Expert Rev. Vaccines. 2018;17(8):677-686. DOI 10.1080/14760584.
2018.1506702.

